# A scoping review of health risks and outcomes from disasters in the Republic of Korea

**DOI:** 10.1186/s12889-025-22362-7

**Published:** 2025-04-11

**Authors:** Dong Ha Kim, Dongjin Kim, Heewon Kang

**Affiliations:** 1 Department of Health Administration, Daejin University, Pocheon-si, Gyeonggi-do 11159 Republic of Korea; 2 Korea Institute for Health and Social Affairs, Sejong-si, 30015 Republic of Korea; 3 Institute of Health and Environment, Seoul National University, 1 Gwanak-ro, Gwanak-gu, Seoul, 08826 Republic of Korea

**Keywords:** Disaster impact, Public health, Risk factors, Vulnerability, Scoping review

## Abstract

**Background:**

Disasters represent significant public health challenges, particularly for vulnerable populations. In the Republic of Korea, both natural and man-made disasters, exacerbated by urbanization and socioeconomic disparities, have exposed weaknesses in disaster preparedness and public health resilience. This scoping review examines health outcomes and associated risk factors from past disasters in Korea.

**Methods:**

A comprehensive search was conducted in the PubMed, DBpia, KISS, and RISS databases for studies published between April 2004 and April 2022, following the PRISMA Extension for Scoping Reviews guidelines. Eighty-three studies met the inclusion criteria. Data were analyzed using a narrative synthesis approach to distinguish direct and indirect health effects. Key outcomes were categorized into socioeconomic, physical, mental, social, and environmental risk factors.

**Results:**

Among the 83 reviewed studies, natural disasters accounted for 50.6% of the total, man-made disasters for 22.9%, and mass trauma events for 26.5%. Most studies (78.3%) focused on disaster survivors, with cross-sectional designs predominating (90.4%). Approximately half (51.8%) of the studies used primary data, with the remainder being based on secondary sources. Regression was the most common method for statistical analysis (75.9%). Frequently reported direct health outcomes included physical injuries such as fractures, burns, and respiratory issues, along with mental health conditions such as post-traumatic stress disorder and depression. Natural disasters were particularly associated with physical injuries, while both natural and man-made disasters had a significant impact on mental health. Vulnerable groups—older adults, women, unmarried individuals, and those with lower socioeconomic status—faced disproportionate higher risk for both physical and mental health. Indirect health impacts such as heightened anxiety, emotional distress, and weakened social cohesion were common in economically disadvantaged and disaster-prone communities, in which recovery was further hindered due to limited access to healthcare and support services.

**Conclusions:**

These findings highlight the need for strategies aimed at disaster risk reduction that prioritize health equity, integrate mental health services, and address environmental vulnerabilities. Future research should focus on longitudinal studies to track evolving health outcomes.

**Supplementary Information:**

The online version contains supplementary material available at 10.1186/s12889-025-22362-7.

## Introduction

Vulnerability to disasters is growing more rapidly than social resilience and response capacity, driven by widespread poverty, unplanned urbanization, and climate change [1]. Disaster risk management is becoming increasingly critical as the frequency and intensity of natural hazards rise. Disaster Risk Reduction (DRR) is a comprehensive approach aimed at reducing disaster vulnerabilities and risks by mitigating both the immediate and long-term health, social, and environmental impacts [2]. Frameworks such as the Sendai Framework for DRR emphasize integrating health outcomes and social determinants into disaster preparedness [2]. However, many countries have not yet fully incorporated health-centered disaster management policies [3]. Global concerns over the rising frequency and impact of disasters underscore the importance of addressing immediate and long-term health vulnerabilities [4, 5].

The World Risk Report estimates that by 2050, 1.5 billion people will live under the constant threat of earthquakes and cyclones [6]. Property damage from disasters has escalated, reaching $140 billion in 2014, and is expected to increase even further in the future [7]. These statistics highlight the urgent need for evidence-based strategies to prevent and/or mitigate the impacts of disasters on health through coordinated global efforts. This calls for a unified, health-centered approach to disaster management that considers regional variations while adhering to international best practices.

Some countries face unique vulnerabilities due to their geographic and geopolitical conditions. For example, the Republic of Korea (hereafter Korea) is exposed to both natural and man-made disasters because of its geographic location and its geopolitical context as a country under armistice. Located within the Pacific Ring of Fire, it faces a heightened risk of earthquakes, tsunamis, and coastal flooding. Moreover, its compact environment and high population density in urban areas, particularly in Seoul, increase vulnerability to outbreaks and rapid spread of infectious diseases such as the 2015 Middle East Respiratory Syndrome (MERS) and Coronavirus Disease 2019 (COVID-19) [8]. A high population density also increases the risk of crowd-related disasters such as the 2022 Itaewon crowd crush. These disasters, which are directly experienced by specific communities and indirectly affect the broader community, reflect vulnerabilities common to rapidly urbanizing and densely populated regions worldwide [3]. These challenges underscore the importance of robust disaster-response frameworks that integrate health vulnerability assessments and are capable of offering valuable lessons for other rapidly urbanizing regions in the globe.

In the aftermath of disasters, the risks of physical injury, loss of life, and exposure to hazardous materials increase [9]. Disruptions to critical services such as access to clean water, food, and healthcare further exacerbate health problems. Integrating disaster preparedness with public health measures is crucial, as observed in past responses to these events [2]. Comparative analyses of how different populations experience health impacts following disasters can help build a resilient global response framework. The COVID-19 pandemic has highlighted and even exacerbated health inequalities, increasing both the direct and peripheral burden on vulnerable populations [10, 11]. Understanding the broader implications of health outcomes following disasters is essential for developing equitable global disaster response strategies [12].

Research is warranted to summarize the available evidence on the health effects of disasters and their relationship with specific regional contexts, such as in the case of Korea. Identifying relevant risk factors for disaster-related health outcomes through international comparisons can guide the development of targeted interventions adapted to different socioeconomic conditions [1, 13]. As global disaster risk management becomes increasingly interconnected with public health, it is crucial to examine how social determinants such as economic disparities and access to healthcare influence health outcomes [10, 11]. Addressing these gaps can contribute to the advancement of disaster-related health management in Korea by offering valuable insights that can be applied to other regions with similar challenges.

This study aimed to systematically review the available evidence on health outcomes and risk factors associated with disasters in Korea to inform both local and global disaster preparedness, response, and recovery strategies. Focusing on the Korean context, the findings aim to offer a broader relevance by providing lessons that can support the development of integrated frameworks applicable to other regions worldwide.

## Methods

### Study design

This study employed a scoping review methodology to systematically map the available literature on health impacts associated with disasters in Korea. Scoping reviews provide an overview of the available evidence on a specific topic, identify gaps in the literature, and guide future research and policymaking [14]. This approach was selected to explore how various types of disasters affect different groups of people, including those directly and indirectly affected. The methodology adhered to the framework established by Arksey and O’Malley [15], and followed the guidelines provided by the PRISMA extension for scoping reviews (PRISMA-ScR) [16], although the protocol for this review was not registered due to time constraints inherent in the commissioned nature of this review.

### Research questions

This review sought to address the following key questions:

1. What are the health impacts of disasters in Korea, and what are the key direct and indirect risk factors associated with these outcomes?
2. How do health impacts differ between direct and indirect victims of the disaster, and what factors influence these differences?
3. How do patterns in disaster-related health outcomes and risk factors differ according to the study design, and what implications do these differences have for understanding the impact of a disaster?

The research questions were designed to capture a comprehensive understanding of disaster-related health outcomes, spanning the physical, mental, and social domains, and to understand how demographic, environmental, and disaster-specific factors shape these outcomes.

### Literature search strategy

A comprehensive search strategy was developed to identify empirical studies in Korea that were published between April 2004 and April 2022 on the health effects of disasters. The year 2004 marked the enactment of the Framework Act on the Management of Disasters and Safety, which established an integrated disaster management system in Korea. Searches were conducted using Korean (DBpia, KISS, and RISS) and international (PubMed) databases. The search terms were informed by disaster definitions from previous studies [17] and by the classifications outlined in the Framework Act on the Management of Disasters and Safety [18]. The search was performed by combining these terms as in the following example: (‘natural disasters’ OR ‘man-made disasters’ OR ‘social disasters’) AND (‘health outcomes’ OR ‘quality of life’) AND (‘Republic of Korea’). “Social disaster” is a term used in the Framework Act on the Management of Disasters and Safety in Korea and includes man-made disasters (e.g., industrial accidents, shootings, acts of terrorism, and incidents of mass violence) as well as incidents of mass trauma (e.g., infectious disease outbreaks, incidents of community unrest, and other types of traumatic events) [19].

### Article screening and eligibility

To ensure the inclusion of relevant studies, a rigorous screening process was conducted. Two independent reviewers (DK and HK) screened the titles and abstracts of all retrieved articles against predefined eligibility criteria. Articles that satisfied the inclusion criteria were then assessed through full-text review. Any disagreements between the reviewers were resolved through a discussion until a consensus was reached. The PRISMA-ScR flow diagram illustrates the inclusion process, which documents the entire selection process from identification to final inclusion.

### Inclusion and exclusion criteria

Studies were included if they focused on health outcomes directly linked to disasters in Korea, encompassing disasters triggered by natural hazards and anthropogenic disaster events. Only peer-reviewed empirical studies utilizing quantitative methods, with a publication window of April 2004 and April 2022, were considered. Full-text availability in English or Korean was required. The exclusion criteria included studies that concentrated solely on disaster preparedness or emergency response without discussing health outcomes, as well as theoretical papers, systematic reviews, editorials, and studies employing qualitative or mixed methods. Additionally, studies relying solely on descriptive statistical analysis without further inferential or analytical interpretation, were excluded. Studies that did not directly focus on Korea were also excluded.

### Data extraction and charting

Data extraction was performed using a standardized form to capture the key characteristics of the included studies. This process involved summarizing the publication year, study location, type of disaster (e.g., earthquake, pandemic, or industrial accident), participant type (e.g., disaster survivors, bereaved families, community residents, or the general population), sample size, research design, data sources, and analytical methods. Health-related outcomes were categorized into physical, mental, and social health and quality of life [20]. Demographic variables such as age, sex, and socioeconomic status were also recorded. This extraction strategy ensured that both direct and indirect health impacts were captured, with data charted in Excel for comparative analysis and identification of patterns and gaps across the studies.

### Data synthesis and analysis

A narrative synthesis approach was used to interpret the extracted data by focusing on content coding and analysis [21]. Content coding involved classifying health-related outcomes into physical, mental, and social domains, including quality of life. We also analyzed socioeconomic, physical, mental, social, and environmental risk factors associated with disaster-related health outcomes. Content analysis identified recurring patterns related to direct and indirect health effects. These included post-disaster mental health issues (e.g., PTSD [post-traumatic stress disorder] or depression) among direct victims, emotional or social consequences among indirect victims, and physical health risks (e.g., injuries or exacerbation of chronic diseases). As this review aimed to provide a comprehensive overview of the existing evidence and to identify gaps in future research, formal quality appraisal was not employed. This review focused instead on identifying thematic patterns and trends rather than placing the emphasis on statistical significance, to ensure a holistic understanding of disaster-related health impacts. All reviewed articles are listed in Supplementary Material 1.

### Ethical considerations

This study involved a review of publicly available literature; thus, no formal ethical approval was required. However, special attention has been paid to the ethical considerations surrounding disaster research, particularly the sensitivity demanded in the study of vulnerable populations such as older adults, people with disabilities, and low-income groups. These ethical considerations were considered in the synthesis of the findings.

## Results

### Study selection

**Fig. 1:**
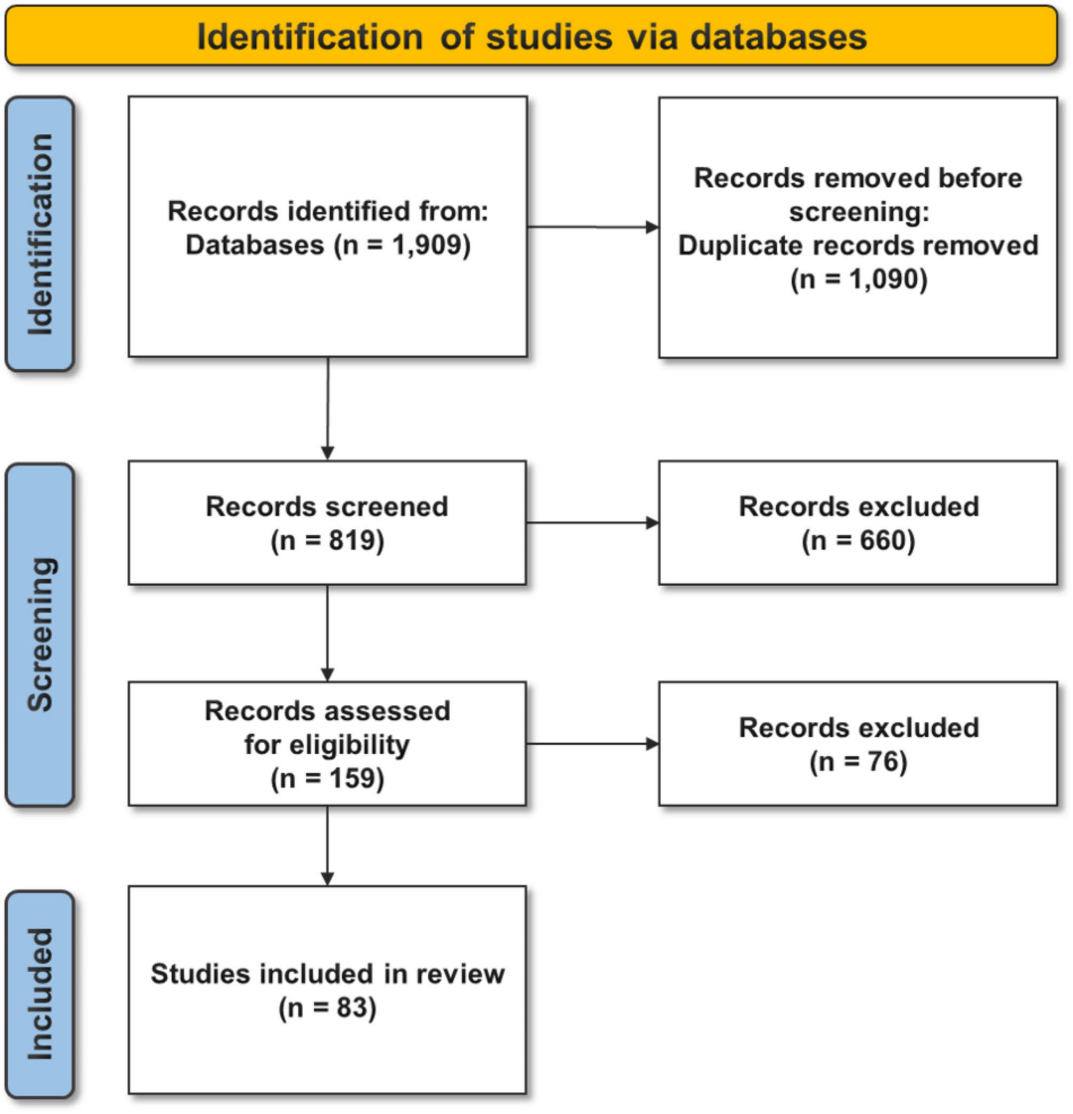
Flow diagram of the study selection

The initial search yielded 1,909 articles, including 392 from PubMed, 857 from RISS, 470 from DBpia, and 190 from KISS. After removing 1,090 duplicates, the titles and abstracts of 819 articles were screened. Of these, 660 were excluded for not meeting the eligibility criteria. The remaining 159 articles underwent full-text evaluation. Subsequently, 76 articles were excluded because they did not meet the specified criteria regarding the focus of the research topic, participants, or outcomes (Fig. 1). A total of 83 articles [22–104] were included in the data extraction and synthesis stage.

### General characteristics of the reviewed articles

**Fig. 2:**
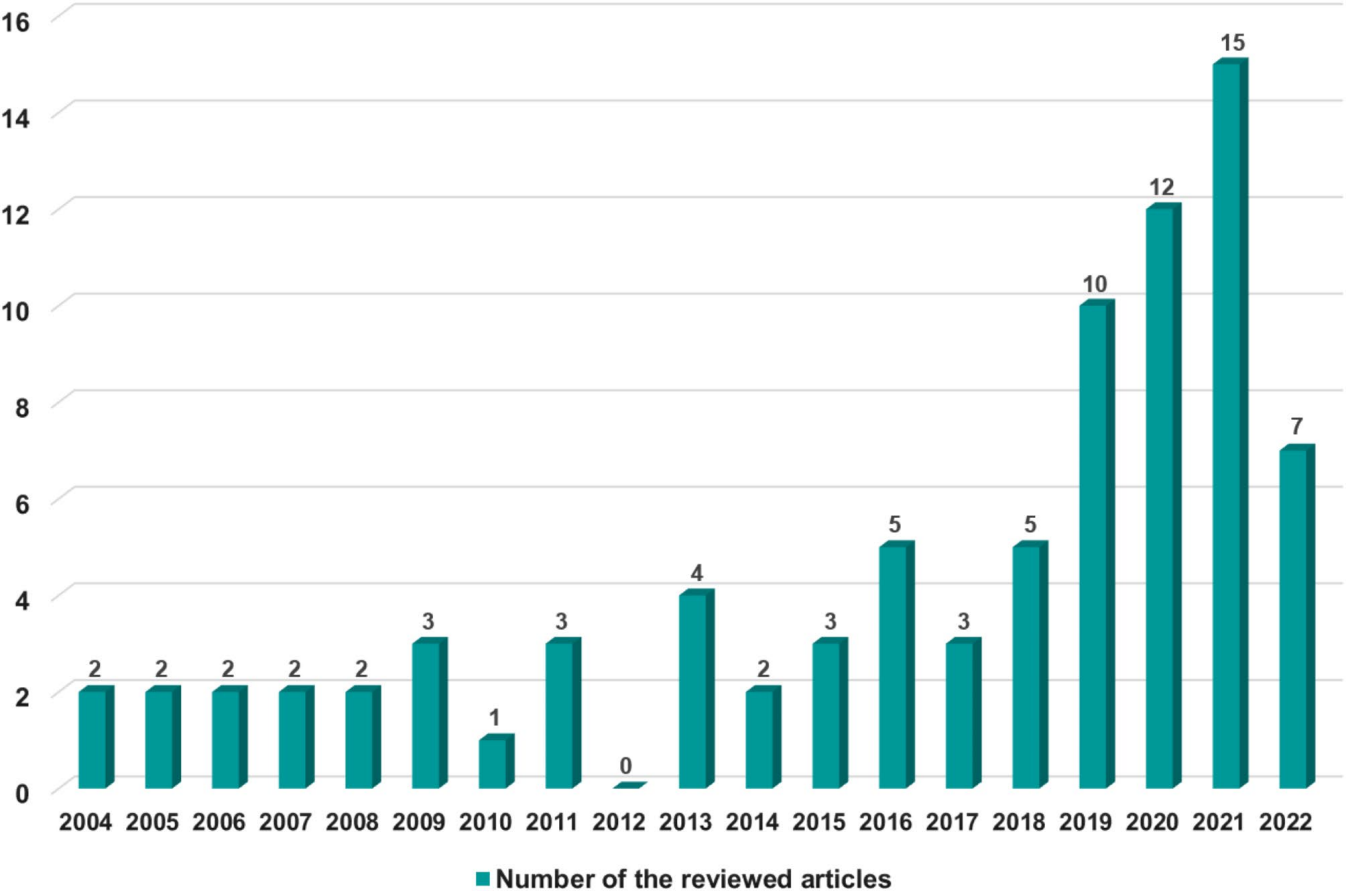
Number of the reviewed articles by year of publication

The number of publications per year ranged from 2 to 5 between 2004 and 2018 but revealed a gradual increase over time during recent years, with 10 articles published in 2019, 12 articles in 2020, and 15 articles in 2021 (Fig. 2).

Regarding disaster types, there were 42 articles (50.6%) on natural hazards [29, 32, 36, 38, 40–50, 56, 57, 61, 64, 66–68, 70–89], 19 articles (22.9%) on man-made disasters [22–28, 30, 31, 33, 35, 52, 90–96], and 22 articles (26.5%) on incidents of mass trauma [34, 37, 39, 50, 53–55, 58–60, 62, 63, 65, 69, 97–104]. The disaster events included geo-meteorological events such as floods, hurricanes, earthquakes, landslides, wildfires, heat waves, or particle pollution (42 articles; 50.6%) [29, 31, 36, 38, 40–49, 51, 56, 57, 61, 64, 66–68, 70–89], infectious disease outbreaks such as those of MERS [34, 58, 98] and COVID-19 [50, 53–55, 59, 62, 63, 65, 69, 102–104] (15 articles; 18.1%), industrial accidents (nine articles; 10.8%) [23, 27, 33, 52, 90, 92, 93, 95, 96], oceanic oil spill accidents (eight articles; 9.6%) [22, 25, 26, 28, 30, 31, 35, 91], mass transportation accidents (seven articles; 8.4%) [37, 39, 60, 97, 99–101], and man-made fire events (two articles; 2.4%) [24, 94].

Most of the articles focused on disaster survivors (*n* = 65, 78.3%). Three articles included disaster survivors as well as bereaved families as study participants [39, 58, 60], and one article included disaster survivors and the general population [61]. Three articles (3.6%) studied residents of the local community who did not suffer direct harm from the disaster but lived in the same spatial area [37, 54, 97], and 11 articles (13.3%) studied the general population [34, 36, 50, 55, 62, 63, 65, 69, 98, 103, 104]. Among the included studies, 75 articles (90.4%) had a cross-sectional design. Seven articles (8.4%) were based on a longitudinal design [28, 44, 52, 73, 74, 79, 80] and one article (1.2%) [76] used a case-crossover design.

In terms of data sources, 43 articles (51.8%) [22–32, 34–36, 39, 50, 53–55, 58–63, 66, 69–72, 84, 86, 90–92, 94, 95, 98–103] were based on primary data collected by the researchers to test specific research questions, while 40 articles (48.2%) [33, 37, 38, 40–49, 51, 52, 56, 57, 64, 65, 67, 68, 73–83, 85, 87–89, 93, 96, 97, 104] used secondary data. A long-term survey of changes in the lives of disaster victims conducted by the Korean National Disaster Management Research Institute (a government-affiliated research body specializing in disaster response and recovery) was the most frequently used secondary data source.

Regression was the most common data analysis method, adopted in 63 articles (75.9%). Twelve articles (14.5%) examined correlations between variables [35, 59, 70–72, 77, 81, 83, 84, 95, 96, 103] and four articles (4.8%) [44, 53, 63, 66] used path analysis or structural equation modeling to examine the relationship between variables. Additionally, four articles (3.6%) employed time series or panel analyses [73, 74, 79, 80] to estimate the relationship between variables using data recorded over time. These findings are summarized in Table 1.

**Table 1:** General characteristics of the reviewed articles (*n* = 83)

Characteristics	Categories	*N* (%)
Types of disasters	Natural disasters Man-made disasters Incidents of mass trauma	42 (50.6) 19 (22.9) 22 (26.5)
Disaster events	Geo-meteorological events Infectious disease outbreaks Industrial accidents Oceanic oil spill accidents Mass transportation accidents Man-made fire events	42 (50.6) 15 (18.1) 9 (10.8) 8 (9.6) 7 (8.4) 2 (2.4)
Participants	Disaster survivors Disaster survivors and bereaved family Disaster survivors and the general population Community residents General population	65 (78.3) 3 (3.6) 1 (1.2) 3 (3.6) 11 (13.3)
Study design	Cross-sectional study Longitudinal study Case-crossover study	75 (90.4) 7 (8.4) 1 (1.2)
Data source	Primary data collection Secondary data collection	43 (51.8) 40 (48.2)
Data analysis	Regression, difference-in-differences, mediation analysis Correlation analysis Time series, panel analysis Structural equation modeling, path analysis	63 (75.9) 12 (14.5) 4 (4.8) 4 (4.8)

### Overview of disaster-related risk factors and health outcomes

**Fig. 3:**
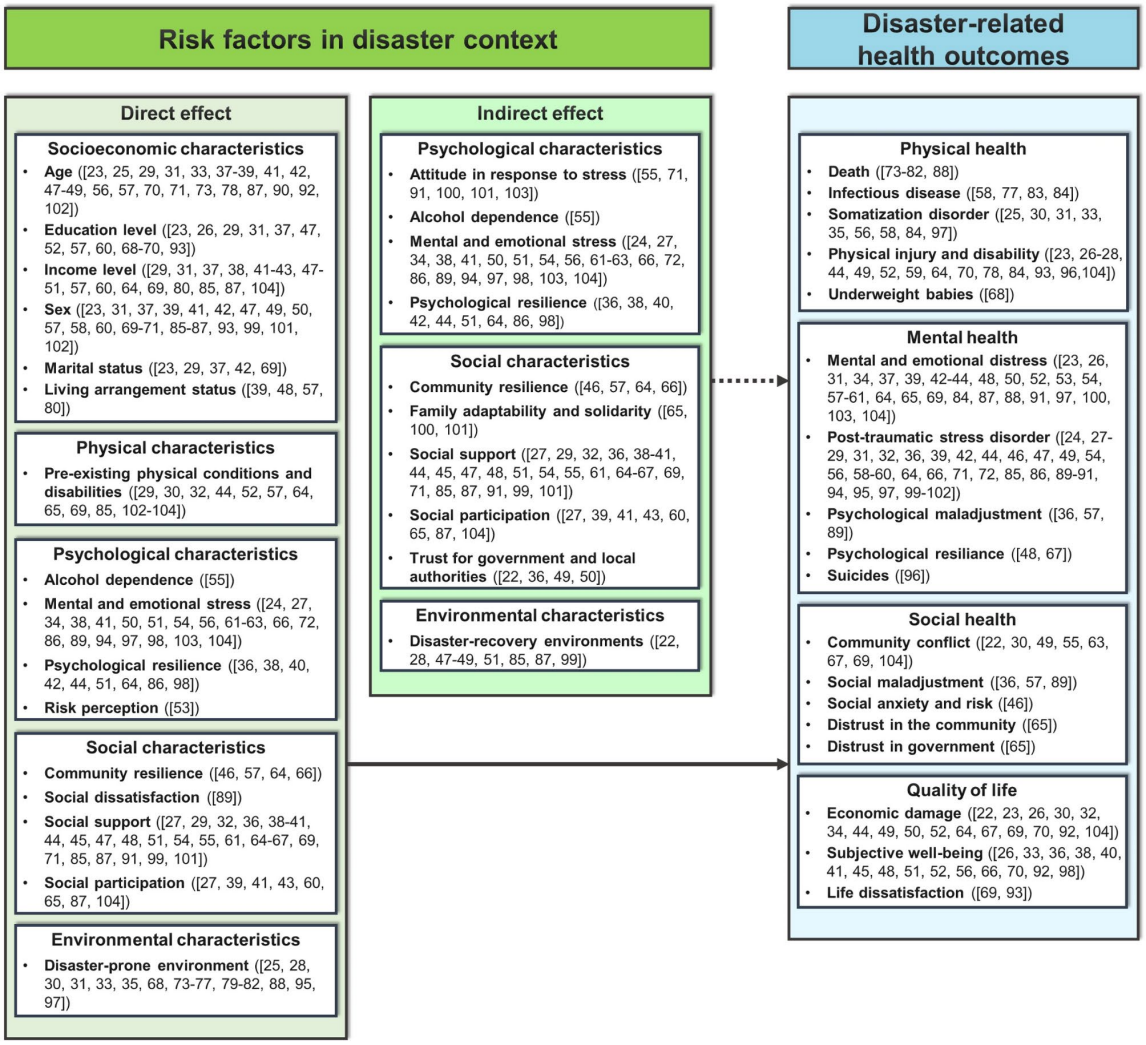
Summary of disaster-related risk factors and health outcomes

Disaster-related health outcomes were influenced by multiple risk factors, categorized into direct and indirect effects (Fig. 3). Direct risk factors include socioeconomic characteristics, pre-existing physical conditions, psychological stress, social dissatisfaction, and disaster-prone environments. Indirect risk factors involve psychological characteristics such as stress response and resilience, social characteristics including community trust and social support, and environmental conditions related to disaster recovery.

These factors contribute to various health outcomes across physical, mental, and social domains, as well as overall quality of life. The following sections examine specific health outcomes and a detailed analysis of risk factors.

### Health effects of disasters identified in the reviewed articles

Table 2 outlines the health outcomes associated with disaster events, distinguishing between groups directly impacted by the event and those only secondarily affected. Direct victims include individuals present at the disaster, while indirect victims encompass family members or broader communities experiencing its repercussions [105, 106].

**Table 2:** Health effects of disasters as identified in the reviewed articles

Categories	Variables	Total (n)	Disaster victims	
			Direct victims (Reference number)	Indirect victims (Reference number)
Physical health	Manifestation of somatization disorder	9	[25, 30, 33, 35, 56, 58, 84, 97]	[31]
	Number of infectious disease cases	4	[58, 77, 83, 84]	
	Level of physical injury and disability	15	[23, 26–28, 44, 49, 52, 59, 64, 70, 78, 84, 93, 96, 104]	
	Number of deaths caused by a disaster	11	[73–82, 88]	
	Number of underweight babies	1	[68]	
Mental health	Level of post-traumatic stress disorder (PTSD)	34	[24, 27–29, 31, 32, 36, 39, 42, 44, 46, 47, 49, 54, 56, 58–60, 64, 71, 72, 85, 86, 89–91, 94, 95, 97, 99–102]	[31, 32, 56, 66]
	Level of mental and emotional distress (depression, anxiety, suicidal ideation)	30	[23, 26, 31, 34, 37, 42–44, 50, 53, 54, 58, 59, 61, 65, 84, 87, 91, 97, 100, 103, 104]	[39, 43, 48, 52, 57, 60, 64, 69, 88, 103]
	Level of psychological resilience	2	[48, 67]	
	Level of psychological maladjustment	3	[57, 89]	[36]
	Number of suicides	1	[96]	
Social health	Degree of community conflict	8	[22, 49, 55, 69, 104]	[30, 63, 67]
	Level of social maladjustment	3	[57, 89]	[36]
	Perception of social anxiety and risk	1		[46]
	level of trust in the community	1		[65]
	Level of trust in government	1		[65]
Quality of life	Level of economic damage	16	[22, 23, 26, 32, 44, 49, 50, 52, 64, 69, 70, 92, 104]	[30, 34, 67]
	Status of subjective well-being	15	[26, 33, 36, 38, 40, 41, 45, 51, 52, 66, 70, 92, 98]	[48, 56]
	Life satisfaction	2	[69, 93]	

For physical health, the identified effects on health include somatization disorder (nine articles) [25, 30, 31, 33, 35, 56, 58, 84, 97], cases of infectious diseases (four articles) [58, 77, 83, 84], physical injuries and disabilities (15 articles) [23, 26–28, 44, 49, 52, 59, 64, 70, 78, 84, 93, 96, 104], deaths caused by a disaster (11 articles) [73–82, 88], and low birth weight (one article) [68]. These physical health outcomes were predominantly reported among direct victims.

Regarding mental health, outcomes such as PTSD (34 articles) [24, 27–29, 31, 32, 36, 39, 42, 44, 46, 47, 49, 54, 56, 58–60, 64, 66, 71, 72, 85, 86, 89–91, 94, 95, 97, 99–102], mental and emotional distress (30 articles) [23, 26, 31, 34, 37, 39, 42–44, 48, 50, 52–54, 57–61, 64, 65, 69, 84, 87, 88, 91, 97, 100, 103, 104], psychological resilience (two articles) [48, 67], psychological maladjustment (three articles) [36, 57, 89], and cases of suicide (one article) [96] were identified. Both direct and indirect victims experienced PTSD, emotional distress, and psychological maladjustment, highlighting the widespread psychological toll of disasters.

Social health outcomes included community conflict (eight articles) [22, 30, 49, 55, 63, 67, 69, 104], social maladjustment (three articles) [36, 57, 89], perception of social anxiety and risk (one article) [46], distrust in the community (one article) [65], and distrust in the government (one article) [65]. Community conflict and social maladjustment were reported among both groups, whereas distrust in institutions was more commonly observed among indirect victims.

For quality of life, key variables included economic damages (16 articles) [22, 23, 26, 30, 32, 34, 44, 49, 50, 52, 64, 67, 69, 70, 92, 104], subjective well-being (15 articles) [26, 33, 36, 38, 40, 41, 45, 48, 51, 52, 56, 66, 70, 92, 98], and life satisfaction (two articles) [69, 93]. These measures captured the effects on both direct and indirect victims, demonstrating the long-term socioeconomic and emotional impacts of disasters.

Health effects varied by study design and the type of disaster victims targeted. Cross-sectional studies primarily examined prevalence-based health outcomes among direct victims, including PTSD, depression, physical symptoms, and quality of life. These studies (75 articles) provided broad snapshots of immediate impacts but did not reflect how symptoms progressed over time or how indirect victims such as family members or emergency responders were affected. Longitudinal studies [28, 44, 52, 73, 74, 79, 80] captured health trajectories, focusing on persistent and compounding risks in both direct and indirect victims. For example, PTSD symptoms in direct victims persisted and intensified over time [44], while prolonged economic instability affected both survivors and their families [52]. Time-series analyses revealed how repeated exposure to heat waves posed a cumulative mortality risk for vulnerable groups, including older adults and low-income populations [73, 74]. These studies effectively assessed delayed and cumulative effects, often missed in cross-sectional research. The only study featuring a case-crossover design [76] focused on acute physiological responses among direct victims, showing that short-term spikes in air pollution were immediately associated with increased cardiovascular mortality. This design was particularly effective for isolating short-term exposure-outcome relationships, emphasizing immediate health risks.

### Risk factors for disaster-related health outcomes

Table 3 shows the results of classifying the risk factors for disaster-related health outcomes by characteristics and reviewing the role of risk factors according to their direct/indirect effects on negative health outcomes. Here, the term “direct effects” is used to denote the immediate impact of disaster-related risk factors on health [107]. On the other hand, the term “indirect effects” is employed to describe situations in which disaster-related risk factors exert mediating or moderating effects on health [108, 109]. By differentiating between direct and indirect effects, we clarified the role of disaster-related risk factors in the pathways leading to health outcomes.

**Table 3:** Risk factors for disaster-related health outcomes as identified in the reviewed articles

Risk factors (direction associated with negative health outcomes)		Role of risk factors	Total (*n*)	Reference number
Socioeconomic characteristics	Age (older)	Direct	23	[23, 25, 29, 31, 33, 37–39, 41, 42, 47–49, 56, 57, 70, 71, 73, 78, 87, 90, 92, 102]
	Sex (female)		22	[23, 31, 37, 39, 41, 42, 47, 49, 50, 57, 58, 60, 69–71, 85–87, 93, 99, 101, 102]
	Income level (lower)		20	[29, 31, 37, 38, 41–43, 47–51, 57, 60, 64, 69, 80, 85, 87, 104]
	Educational level (lower)		13	[23, 26, 29, 31, 37, 47, 52, 57, 60, 68–70, 93]
	Marital status (single)		5	[23, 29, 37, 42, 69]
	Living arrangement status (single-person household)		4	[39, 48, 57, 80]
Physical characteristics	Pre-existing physical conditions and disabilities (with medical history)	Direct	13	[29, 30, 32, 44, 52, 57, 64, 65, 69, 85, 102–104]
Psychological characteristics	Mental and emotional stress (depression, anxiety, fear, helplessness, suicidal thoughts) (higher)	Direct, indirect	21	[24, 27, 34, 38, 41, 50, 51, 54, 56, 61–63, 66, 72, 86, 89, 94, 97, 98, 103, 104]
	Psychological resilience (lower)	Direct, indirect	9	[36, 38, 40, 42, 44, 51, 64, 86, 98]
	Attitude in response to stress (negative, evasive)	Indirect	6	[55, 71, 91, 100, 101, 103]
	Risk perception (higher)	Direct	1	[53]
	Alcohol dependence (stronger)	Direct, indirect	1	[55]
Social characteristics	Social support (lower)	Direct, indirect	27	[27, 29, 32, 36, 38–41, 44, 45, 47, 48, 51, 54, 55, 61, 64–67, 69, 71, 85, 87, 91, 99, 101]
	Social participation (lower)	Direct, indirect	8	[27, 39, 41, 43, 60, 65, 87, 104]
	Family adaptability and solidarity (lower)	Indirect	3	[65, 100, 101]
	Trust for government and local authorities (lower)	Indirect	4	[22, 36, 49, 50]
	Community resilience (lower)	Direct, indirect	4	[46, 57, 64, 66]
	Social dissatisfaction (higher)	Direct	1	[89]
Environmental characteristics	Disaster-prone environment (vulnerable)	Direct	19	[25, 28, 30, 31, 33, 35, 68, 73–77, 79–82, 88, 95, 97]
	Disaster-recovery environments (less)	Indirect	9	[22, 28, 47–49, 51, 85, 87, 99]

#### Socioeconomic characteristics

The socioeconomic characteristics influencing disaster-related health outcomes included age (23 articles) [23, 25, 29, 31, 33, 37–39, 41, 42, 47–49, 56, 57, 70, 71, 73, 78, 87, 90, 92, 102], sex (22 articles) [23, 31, 37, 39, 41, 42, 47, 49, 50, 57, 58, 60, 69–71, 85–87, 93, 99, 101, 102], income level (20 articles) [29, 31, 37, 38, 41–43, 47–51, 57, 60, 64, 69, 80, 85, 87, 104], education level (13 articles) [23, 26, 29, 31, 37, 47, 52, 57, 60, 68–70, 93], marital status (five articles) [23, 29, 37, 42, 69], and living arrangement (four articles) [39, 48, 57, 80].

The results suggest that individuals who are older, female, unmarried, living alone, or have lower income and education levels are more vulnerable to adverse health outcomes in the context of disasters. These characteristics function as direct risk factors and increase susceptibility to adverse health effects during and after disasters.

#### Physical characteristics

Pre-existing physical conditions and disabilities (e.g., cardiovascular diseases, respiratory illnesses, diabetes, sleep problems, and mobility impairments) (13 articles) [29, 30, 32, 44, 52, 57, 64, 65, 69, 85, 102–104] were identified as physical characteristics directly associated with increased vulnerability to negative health outcomes in disasters. Individuals with such conditions are at higher risk of adverse health effects when disasters occur.

#### Psychological characteristics

Psychological characteristics that affected disaster-related health outcomes included mental and emotional stress (21 articles) [24, 27, 34, 38, 41, 50, 51, 54, 56, 61–63, 66, 72, 86, 89, 94, 97, 98, 103, 104], psychological resilience (nine articles) [36, 38, 40, 42, 44, 51, 64, 86, 98], attitude in response to stress (six articles) [55, 71, 91, 100, 101, 103], risk perception (one article) [53], and alcohol dependence (one article) [55].

Higher levels of psychological distress, high risk perception, alcohol use, and negative attitudes towards stress (such as avoidance or denial) are associated with negative disaster-related health outcomes. Conversely, low psychological resilience has been linked to poor health outcomes [36, 38, 40, 42, 44, 51, 64, 86, 98]. These psychological factors act not only as direct determinants of health outcomes but also as mediators. Mental and emotional stress, psychological resilience, and attitudes towards stress mediated the relationship between disaster exposure and health impacts, with mental and emotional stress having both direct and indirect effects.

#### Social characteristics

Social characteristics that influenced disaster-related health outcomes included social support (27 articles) [27, 29, 32, 36, 38–41, 44, 45, 47, 48, 51, 54, 55, 61, 64–67, 69, 71, 85, 87, 91, 99, 101], social participation (eight articles) [27, 39, 41, 43, 60, 65, 87, 104], trust in the government and local authorities (four articles) [22, 36, 49, 50], community resilience (four articles) [46, 57, 64, 66], family adaptability and cohesion (three articles) [65, 100, 101], and social dissatisfaction (one article) [89].

The direction of these risk factors indicates that lower levels of social support, social participation, family solidarity and adaptability, community resilience, and trust in the government and local authorities, as well as higher social dissatisfaction were associated with increased adverse health outcomes during disasters. Specifically, low social support, reduced social participation, and decreased community resilience were found to have both direct and indirect effects, indicating that they directly affected health outcomes and mediated the relationship between disaster exposure and health consequences [29, 32, 36, 38–41, 44, 45, 47, 48, 51, 54, 55, 61, 64–67, 69, 71, 85, 87, 91, 99, 101]. On the other hand, family solidarity and adaptability, trust in the government and local authorities, and social dissatisfaction were only associated with indirect effects, indicating that they influence health outcomes by altering other factors mediating the impact of the disaster [22, 36, 49, 50, 65, 89, 100, 101].

#### Environmental characteristics

Environmental characteristics that influenced disaster-related health outcomes were included disaster-prone environments (e.g., neighborhoods with poor access to infrastructure, medical facilities, or emergency services; those at high risk of natural disasters, or regions particularly vulnerable to extreme heat events) (19 articles) [25, 28, 30, 31, 33, 35, 68, 73–77, 79–82, 88, 95, 97] and capacity of the environment for recovery (nine articles) [22, 28, 47–49, 51, 85, 87, 99]. The more vulnerable the environment to which disaster victims are exposed, such as areas with insufficient infrastructure or resources, the greater the adverse health outcomes associated with disaster events. Similarly, environments where recovery from disaster is less effective due to inadequate relief services or slow community recovery are associated with worse health outcomes [22, 28, 47–49, 51, 85, 87, 99].

Disaster-prone environments act as direct risk factors, implying that they immediately impact the health of those affected by disasters. Conversely, the ability of an environment to recover from disaster has an indirect role as a moderating variable, influencing the extent and severity of adverse health outcomes by affecting the post-disaster recovery process [22, 28, 47–49, 51, 85, 87, 99]. These findings highlight the dual role of environmental characteristics in determining the immediate and long-term health impacts of disasters.

#### Study design and patterns in risk factors

Risk factors varied according to the study design and the type of effects (direct vs. indirect) examined. Cross-sectional studies primarily identified point-in-time correlations with direct effects, such as socioeconomic vulnerabilities (e.g., low income or education levels) and physical characteristics (e.g., pre-existing conditions) [23, 26, 29, 31, 37, 39, 41, 43, 47, 49]. These studies offered overviews of disaster-related risks and frequently highlighted immediate impacts, such as the higher susceptibility of older adults, women, and single-person households to disaster-related health risks [25, 29, 31, 33, 35, 37, 39, 41, 42, 47, 49]. However, they provided limited insight into how these disparities evolved over time or indirectly affected the broader population, including family members or communities experiencing secondary stressors [22, 24, 36, 38, 44, 50, 64].

In contrast, longitudinal studies [28, 44, 52, 73, 74, 79, 80] captured progressive and cumulative risks, encompassing both direct and indirect effects. For example, economic instability among disaster survivors not only persisted post-disaster but also extended to their households, indirectly affecting family well-being and long-term recovery [52]. Similarly, prolonged air pollution exposure was shown to increase the burden of chronic disease in vulnerable populations, highlighting compounding risks over time [79]. Studies on the effects of heat waves showed a cumulative mortality risks among older adults and low-income groups, emphasizing how vulnerabilities were intensified with repeated exposures [73, 74]. These studies were particularly effective in addressing indirect risks, such as the erosion of social cohesion or prolonged psychological distress, which often remain unaddressed in short-term analyses.

The case-crossover study [76] focused exclusively on direct effects, particularly acute environmental risk factors. It demonstrated that short-term spikes in air pollution immediately increased cardiovascular mortality, providing critical insights into time-sensitive risk factors. This approach was uniquely suited for isolating immediate physiological responses to acute exposures, minimizing confounding variables associated with indirect, long-term effects.

## Discussion

This scoping review encompasses a wide range of disaster-related health impacts in Korea, including not only immediate physical injuries and long-term mental health effects but also broader effects on quality of life, social well-being, and economic conditions.

In Korea, the increasing trend in disaster research highlights the growing awareness of the complexities of disaster impact. However, the reliance on cross-sectional designs limits the ability to evaluate long-term outcomes. Future research should prioritize longitudinal studies that track participants over time, particularly to understand the evolution of the impact on health, including chronic conditions and psychological resilience. Expanding the scope to include not only the immediate victims but also communities indirectly affected by the disaster will provide a more comprehensive understanding of its consequences [108]. Standardized data collection and advanced analytical methods such as longitudinal modeling and panel analysis are critical to enhance the reliability of the findings. These methods enable more robust comparisons of DRR across countries, demonstrating how different socioeconomic and policy factors influence disaster preparedness and outcomes, and providing strategies that could be adopted by Korea to enhance its resilience [2]. Such approaches will strengthen the policies currently in place and also contribute to global efforts towards enhanced disaster resilience.

Our results revealed a predominant focus on direct disaster victims, particularly in terms of physical and mental health outcomes, with less attention given to indirect victims such as families and communities affected by secondary means. This limited scope highlights a gap in our understanding of the long-term and broader psychological and social impact of disasters. While PTSD and emotional distress are frequently examined, the underrepresentation of social outcomes such as community conflict and distrust in institutions suggests that disaster recovery efforts often overlook the importance of rebuilding social cohesion [110]. Furthermore, current assessments of disaster recovery rely heavily on economic damage estimates and subjective well-being indicators, underscoring the need for more diverse metrics, such as long-term life satisfaction and community resilience [111]. To address these gaps, DRR strategies must prioritize mental health interventions and include comprehensive support for both direct and indirect victims, while expanding the metrics used to measure recovery to ensure a holistic, long-term approach to community restoration beyond immediate physical recovery.

This review highlights the influence of socioeconomic status on health outcomes resulting from disasters, particularly in low-income groups and in those with limited access to resources. Socioeconomic inequalities increase vulnerability to both direct effects, such as physical injuries, and indirect consequences, such as prolonged psychological distress and social isolation. Individuals with lower income, lower educational attainment, or those living alone are more susceptible to these risks due to delays in accessing emergency services and inadequate financial resources [21, 24, 27, 29, 35–37, 39–41, 45–50, 55, 58, 62, 66–68, 78, 83, 85, 91]. For instance, one of the reviewed studies found that low-income households affected by floods in Korea experienced significantly longer recovery times and a higher economic burden, as limited resources delayed housing repairs and healthcare access [27, 35]. Similarly, older adults living alone exhibited higher rates of PTSD due to social isolation and insufficient community support networks [66].

Addressing these inequalities requires integrating disaster assistance into broader social protection frameworks to prevent long-term economic instability and minimize unmet needs. The COVID-19 pandemic showed significant deficits in disaster relief programs, particularly those targeting marginalized groups such as homeless individuals, migrants, and irregular workers, underscoring the overlap between limitations in disaster support and pre-existing welfare blind spots [111]. Streamlining administrative processes and enhancing outreach efforts can reduce barriers for vulnerable populations and promote a more inclusive recovery. Tailored interventions, such as employment stability programs and accessible healthcare services, are essential to minimize health and economic disparities during crises. Ensuring equitable access to essential resources, such as healthcare delivery systems, emergency relief supplies, and public health programs, is key to building resilience [111]. International examples provide valuable models: Evacuation plans for older adults and individuals with disabilities in Japan emphasize accessibility and tailored support [112], while the whole-community approach of the Federal Emergency Management Agency integrates marginalized groups into recovery planning and financial aid distribution [113]. Similarly, the Student Volunteer Army from New Zealand demonstrates the effectiveness of community-based initiatives in strengthening social networks during recovery [114]. These efforts, combined with targeted social policies, can mitigate both the immediate physical and long-term psychological impacts of disasters.

Preexisting physical conditions and disabilities significantly influence disaster-related health outcomes. Individuals with physical vulnerabilities face higher risks during disasters owing to limitations in mobility and access to healthcare services [1]. For example, after the 2023 Turkey–Syria earthquake, people with disabilities reported significant barriers to evacuation, access to emergency shelters, and a lack of tailored preparedness measures, highlighting the vulnerability of this population to major disasters [115]. These direct barriers often lead to indirect effects such as prolonged recovery times and heightened psychological stress, which can exacerbate the overall health burden on vulnerable groups. Moreover, additional difficulties in accessing healthcare services post-disaster can intensify pre-existing conditions, indirectly compounding health vulnerabilities over time. These findings align with international research that stresses the importance of disability-inclusive disaster management strategies [116]. Ensuring that disaster preparedness frameworks, especially regarding DRR policies, are disability-inclusive can significantly improve outcomes for people with disabilities. Accessible communication systems, evacuation protocols that take different physical abilities into account, and healthcare services designed to address the specific needs of this population should be integrated into DRR policies [111]. Involving people with disabilities in the development of these frameworks ensures that their needs are fully understood and addressed. Disaster preparedness drills and public awareness campaigns should include disability-inclusive strategies to improve resilience. Such inclusive strategies can help address both the immediate physical risks and the secondary social and mental health effects of disasters.

Social capital and community resilience have emerged as essential factors for disaster recovery. Communities with robust social networks are better able to respond to and recover from disasters due to the support they provide [117]. This applies not only to addressing direct impacts such as immediate recovery efforts, but also to mitigating indirect effects such as long-term social fragmentation and psychological stress. When social networks are disrupted, indirect consequences such as feelings of isolation or lack of trust in the community can hinder both mental and social recovery. The Hyogo Framework for Action (2005–2015) and Sendai Framework for DRR (2015–2030) recognize social capital as a vital resource for building community resilience and encourage governments to invest in community-based DRR strategies [118]. However, many countries, including Korea, face significant challenges in implementing practical programs aimed to building social capital during the disaster preparedness and recovery phases [111]. Incorporating community-based DRR strategies that focus on social capital can significantly improve resilience at the local level, ensuring substantial disaster recovery efforts. Such strategies can mitigate immediate physical damage and address long-term indirect impacts such as the erosion of trust in the community and in government institutions [117]. Investing in initiatives that strengthen social capital, such as community engagement programs and disaster preparedness education, can significantly improve the ability of communities to withstand and recover from disasters. Fostering collaboration between governmental and non-governmental organizations would further enhance DRR efforts by creating a more resilient and interconnected social fabric.

Environmental factors significantly influence disaster-related health outcomes, especially in regions prone to recurrent natural hazards such as floods and landslides. Populations in these high-risk areas face growing vulnerabilities, making it essential to establish specialized disaster management bodies focused on prevention, risk assessment, and mitigation strategies. This review highlights the need for dedicated institutions modeled after those in Japan, the United States, and European countries such as Germany to examine the causes of disasters and implement evidence-based interventions to prevent their recurrence [119]. Regular disaster risk assessments and monitoring of environmental vulnerabilities in high-risk areas are crucial for the success of preventive efforts. Institutional frameworks should include mechanisms to generate detailed disaster statistics. Currently, disaster statistics in Korea are fragmented across multiple government agencies, limiting their ability to track the impact on at-risk groups [111]. The lack of centralized data exacerbates the indirect effects of disasters by slowing down recovery efforts and prolonging vulnerability in affected populations. A centralized system that collects comprehensive data on physical, economic, and social recovery outcomes is essential to formulate fact-based policies and tailor DRR strategies to the specific needs of these regions. Integrating disaster monitoring systems across government agencies can improve the accuracy of impact tracking and ensure that DRR policies address environmental and social vulnerabilities [3]. Aligning the disaster management strategy in Korea with international best practices, such as the Civil Protection Mechanism implemented by the European Union [120], would enhance resilience and position Korea as a model for other nations facing similar challenges, fostering domestic and global disaster preparedness.

Patterns in disaster-related health effects and risk factors differed according to the study design, shaping the scope of the findings and their implications. Our analysis showed that cross-sectional studies focused on immediate direct effects, while longitudinal studies captured persistent and indirect risks, and case-crossover studies identified acute, time-sensitive risks. These differences reflect the unique strengths and limitations of each study design in capturing disaster-related health outcomes. The interpretation of these findings is constrained by the availability and structure of the disaster-related data. Additionally, the lack of standardized, longitudinal datasets can pose challenges in the analysis of how chronic conditions, psychological distress, and socioeconomic disparities evolve post-disaster [121]. This limitation is further compounded by the predominant focus on direct victims in disaster research, with indirectly affected populations receiving considerably less attention [122]. To develop a more comprehensive understanding of the phenomenon, future research should prioritize long-term health monitoring systems, panel datasets, and multi-dimensional approaches [3]. These methods should integrate both direct and indirect impacts of the disaster.

### Limitations

This study had several limitations. First, there is the potential for reporting bias, as studies that report statistically significant outcomes are more likely to be published than those with negative results. This bias can result in overestimation of the health impact of disasters [123]. Secondly, the review was limited to studies published in peer-reviewed journals and available in English or Korean, which may have excluded relevant studies published in other languages. Third, most studies included in this review utilized cross-sectional designs, which limited the ability to establish causality between disaster exposure and health outcomes. Additionally, the heterogeneity of study designs and outcome measures limited the comparability of results, particularly regarding effect sizes. This review also highlights that focusing solely on statistically significant findings may overlook important context-specific risk factors, further restricting the understanding of the phenomenon. A formal comparison of effect sizes and statistical significance across studies was not conducted, as this fell outside the primary objectives of a scoping review, which prioritizes the identification of broader patterns and thematic insights. Finally, since the review focused on Korea, the findings may not be generalizable to other regions with different geographic, socioeconomic, and cultural contexts.

## Conclusions

This scoping review highlights opportunities to strengthen DRR efforts, both globally and specifically in Korea, by integrating health equity into disaster preparedness strategies. Supporting socioeconomically disadvantaged groups and individuals with pre-existing conditions is critical, given the cascading and long-term impacts beyond immediate physical harm. Incorporating mental health services and socioeconomic support into DRR frameworks can enhance resilience, while localized interventions addressing specific social and environmental vulnerabilities remain essential. The experience of Korea offers insight into broader disaster health management, which is particularly important in areas where research remains limited in scope, indirect victims are underrepresented, and long-term health effects are not sufficiently examined. Given the multifaceted and compounding nature of disasters, there is a need to shift toward longitudinal health monitoring, community-based frameworks, and integrated approaches that connect immediate response with long-term recovery. Strengthening international collaboration through standardized data collection and cross-national studies will contribute to evidence-based policymaking and enhanced global disaster resilience.

## Electronic supplementary material

Below is the link to the electronic supplementary material.

Supplementary Material 1

## Data Availability

All data relevant to the study are included in the article or have been uploaded as supplementary information. Any additional datasets generated and/or analyzed during the study are available from the corresponding author upon reasonable request.

## Abbreviations

COVID-19: Coronavirus Disease 2019
DRR: Disaster Risk Reduction
MERS: Middle East Respiratory Syndrome
PRISMA-ScR: PRISMA extension for scoping reviews
PTSD: Post-Traumatic Stress Disorder
