# Supplementary material for: A scoping review of health risks and outcomes from disasters in the Republic of Korea
Source: BMC Public Health. 2025 Apr 11;25:1369. doi: 10.1186/s12889-025-22362-7 (PMC11987192; doi:10.1186/s12889-025-22362-7)
Supplement: Supplementary file 1 — Supplementary Material 1 [file 12889_2025_22362_MOESM1_ESM.docx]

**Supplementary 1**. General characteristics of the reviewed articles

| **Author (year)** | **Disaster types** | **Disaster events** | **Participants** | **Sample size** | **Study design** | **Data source** | **Analysis** |
| --- | --- | --- | --- | --- | --- | --- | --- |
| [1] Lee (2004) | Natural | Floods, hurricanes | Disaster victims  (adults) | 248 | Cross-sectional | Primary | Correlation |
| [2] Lee et al. (2004) | Natural | Floods, hurricanes | Disaster victims  (elementary students) | 261 | Cross-sectional | Primary | Correlation |
| [3] Chae et al. (2005) | Natural | Floods, hurricanes | Disaster victims  (middle/high school students, adults) | 585 | Cross-sectional | Primary | Correlation |
| [4] Woo et al. (2005) | Man-made | Industrial accidents | Disaster victims  (adults) | 628 | Cross-sectional | Primary | Regression |
| [5] Kim et al. (2006) | Natural | Heat wave | Disaster victims  (adults) | 1,442 | Longitudinal | Secondary | Time series |
| [6] Park & Kim (2006) | Man-made | Industrial accidents | Disaster victims  (adults) | 206 | Cross-sectional | Primary | Regression |
| [7] Lee D.-G. et al. (2007) | Natural | Heat wave | Disaster victims  (adults) | 720 | Longitudinal | Secondary | Time series |
| [8] Lee E. J. et al. (2007) | Man-made | Fire  (Daegu subway fire) | Disaster victims  (adults) | 49 | Cross-sectional | Primary | Regression |
| [9] Kim & Kwon (2008) | Man-made | Environmental pollution  (Hebei Spirit oil spill) | Disaster victims  (adults) | 464 | Cross-sectional | Primary | Regression |
| [10] Park & Kim (2008) | Man-made | Industrial accidents | Disaster victims  (adults) | 195 | Cross-sectional | Primary | Regression |
| [11] Chang & Choi (2009) | Natural | Fine dust | Disaster victims  (adults) | Not reported | Cross-sectional | Secondary | Regression |
| [12] Kim et al. (2009) | Man-made | Environmental pollution  (Hebei Spirit oil spill) | Disaster victims  (Pregnant women) | 80 | Cross-sectional | Primary | Regression |
| [13] Song et al. (2009) | Man-made | Environmental pollution  (Hebei Spirit oil spill) | Disaster victims  (adults) | 71 | Cross-sectional | Primary | Regression |
| [14] Lee et al. (2010) | Man-made | Environmental pollution  (Hebei Spirit oil spill) | Disaster victims  (adults) | 299 | Cross-sectional | Primary | Regression |
| [15] Choi et al. (2011) | Man-made | Industrial accidents | Disaster victims  (adults) | 491 | Cross-sectional | Primary | Regression |
| [16] Ha et al. (2011) | Natural | Fine dust | Disaster victims  (adults) | 160,273 | Case-cross over | Secondary | Regression |
| [17] Lee & Kim (2011) | Man-made | Environmental pollution  (Hebei Spirit oil spill) | Disaster victims  (adults) | 185 | Longitudinal | Primary | Regression |
| [18] Cho & Yang (2013) | Natural | Floods | Disaster victims  (adults) | 148 | Cross-sectional | Primary | Regression |
| [19] Kim et al. (2013) | Natural | Floods, hurricanes | Disaster victims  (adults) | Not reported | Cross-sectional | Secondary | Correlation |
| [20] Lee et al. (2013) | Natural | Floods, hurricanes, landslides | Disaster victims  (adults) | 642 | Cross-sectional | Secondary | Regression |
| [21] Park & Sim (2013) | Man-made | Environmental pollution  (Hebei Spirit oil spill) | Disaster victims  (adults) | 763 | Cross-sectional | Primary | Regression |
| [22] Bae (2014) | Natural | Fine dust | Disaster victims  (adults) | 43,063 | Longitudinal | Secondary | Time series |
| [23] Lee et al. (2014) | Natural | Heat wave | Disaster victims  (older adults) | Not reported | Longitudinal | Secondary | Panel |
| [24] Park et al. (2015) | Natural | Fine dust | Disaster victims  (adults) | 3,732 | Cross-sectional | Secondary | Correlation |
| [25] Shin et al. (2015) | Natural | Heat wave | Disaster victims  (older adults) | 1,320 | Cross-sectional | Secondary | Regression |
| [26] Yang et al. (2015) | Incidents of mass trauma | Transport disaster  (sinking of Sewol Ferry) | Community residents  (adults) | 7,076 | Cross-sectional | Secondary | Regression |
| [27] Choi K. H. et al. (2016) | Man-made | Environmental pollution  (Hebei Spirit oil spill) | Disaster victims  (adults) | 993 | Cross-sectional | Primary | Regression |
| [28] Choi W.S. et al. (2016) | Man-made | Industrial accidents | Disaster victims  (adults) | 1,832 | Cross-sectional | Secondary | Regression |
| [29] Kim (2016) | Man-made | Industrial accidents | Disaster victims  (adults) | 5,816 | Cross-sectional | Secondary | Regression |
| [30] Lee et al. (2016) | Incidents of mass trauma | Infectious disease outbreaks  (MERS) | General population  (adults) | 450 | Cross-sectional | Primary | Regression |
| [31] Na et al. (2016) | Natural | Floods, hurricanes | Disaster victims  (adults) | 312 | Cross-sectional | Secondary | Correlation |
| [32] Hong et al. (2017) | Natural | Hurricanes | Disaster victims  (adults) | 600 | Cross-sectional | Primary | Correlation |
| [33] Kim et al. (2017) | Man-made | Environmental pollution  (Hebei Spirit oil spill) | Disaster victims  (adults) | 628 | Cross-sectional | Primary | Correlation |
| [34] Kwon et al. (2017) | Incidents of mass trauma | Infectious disease outbreaks  (MERS) | General population  (adults) | 127 | Cross-sectional | Primary | Mediation |
| [35] Lee S. H. et al. (2018a) | Incidents of mass trauma | Transport disaster  (sinking of Sewol Ferry) | Disaster victims  (survivors) | 57 | Cross-sectional | Primary | Regression |
| [36] Lee S. H. et al. (2018b) | Incidents of mass trauma | Transport disaster  (sinking of Sewol Ferry) | Disaster victims  (survivors) | 57 | Cross-sectional | Primary | Regression |
| [37] Lim & Sim (2018) | Natural | Floods, hurricanes | Disaster victims  (adults) | 1,182 | Cross-sectional | Primary | Regression |
| [38] Min et al. (2018) | Natural | Earthquake  (2016 Gyeongju earthquake) | General population  (adults) | 322 | Cross-sectional | Primary | Regression |
| [39] Yang et al. (2018) | Incidents of mass trauma | Transport disaster  (sinking of Sewol Ferry) | Community residents  (adults) | 3,521 | Cross-sectional | Secondary | Regression |
| [40] Cho (2019) | Natural | Floods, hurricanes, earthquakes, wildfires | Disaster victims  (adults) | 1,659 | Cross-sectional | Secondary | Regression |
| [41] Han et al. (2019) | Incidents of mass trauma | Transport disaster  (sinking of Sewol Ferry) | Disaster victims (survivors),  Bereaved family | 241 | Cross-sectional | Primary | Regression |
| [42] Jeon et al. (2019) | Man-made | Fire  (Jecheon sports center fire) | Disaster victims  (adults) | 42 | Cross-sectional | Primary | Regression |
| [43] Kim & Kim (2019) | Natural | Floods, hurricanes, earthquakes, wildfires | Disaster victims  (adults) | 1,659 | Cross-sectional | Secondary | Mediation |
| [44] Kim & Lee (2019) | Natural | Floods, hurricanes, earthquakes | Disaster victims  (adults) | 572 | Cross-sectional | Secondary | Regression |
| [45] Kim & Oh (2019) | Natural | Earthquake  (2016 Gyeongju earthquake) | Disaster victims  (adults) | 493 | Cross-sectional | Primary | Regression |
| [46] Kim (2019) | Natural | Floods, hurricanes, earthquakes, wildfires | Disaster victims  (older adults) | 666 | Cross-sectional | Secondary | Regression |
| [47] Kim et al. (2019) | Natural | Floods, hurricanes, earthquakes, wildfires | Disaster victims  (older adults) | 666 | Cross-sectional | Secondary | Regression |
| [48] Noh et al. (2019) | Incidents of mass trauma | Transport disaster  (sinking of Sewol Ferry) | Disaster victims  (survivors) | 57 | Cross-sectional | Primary | Regression |
| [49] Sim (2019) | Natural | Floods, hurricanes, earthquakes, wildfires | Disaster victims  (middle/high school students, adults) | 353 | Cross-sectional | Secondary | Regression |
| [50] Choi (2020) | Natural | Floods, hurricanes, earthquakes, wildfires | Disaster victims  (older adults) | 666 | Cross-sectional | Secondary | Regression |
| [51] Kim & Kim (2020) | Natural | Floods, hurricanes, earthquakes, wildfires | Disaster victims  (adults) | 464 | Longitudinal | Secondary | Structural equation modeling |
| [52] Kim & Lee (2020) | Natural | Floods, hurricanes, earthquakes, wildfires | Disaster victims  (single-person household) | 196 | Cross-sectional | Secondary | Regression |
| [53] Kim & Oh (2020) | Natural | Floods, hurricanes, earthquakes, wildfires | Disaster victims  (adults) | 2,311 | Cross-sectional | Secondary | Regression |
| [54] Kim S. Y. (2020) | Natural | Floods, hurricanes, earthquakes, wildfires | Disaster victims  (adults) | 2,311 | Cross-sectional | Secondary | Regression |
| [55] Kim S. (2020) | Natural | Floods, hurricanes, earthquakes, wildfires | Disaster victims  (adults) | 1,609 | Cross-sectional | Secondary | Regression |
| [56] Lee N. et al. (2020) | Natural | Floods, hurricanes, earthquakes, wildfires | Disaster victims  (adults) | 1,390 | Cross-sectional | Secondary | Regression |
| [57] Lee D. -H. et al. (2020) | Incidents of mass trauma | Infectious disease outbreaks  (COVID-19) | General population  (adults) | 600 | Cross-sectional | Primary | Regression |
| [58] Lim et al. (2020) | Man-made | Industrial accidents | Disaster victims  (adults) | 125 | Cross-sectional | Primary | Correlation |
| [59] Park & Chae (2020) | Natural | Heat wave | Disaster victims  (adults) | 44,989 | Cross-sectional | Secondary | Regression |
| [60] Song & Choi (2020) | Natural | Floods, hurricanes, earthquakes, wildfires | Disaster victims  (older adults) | 666 | Cross-sectional | Secondary | Regression |
| [61] Song et al. (2020) | Natural | Floods, hurricanes, earthquakes, wildfires | Disaster victims  (older adults) | 666 | Cross-sectional | Secondary | Regression |
| [62] Bae et al. (2021) | Man-made | Industrial accidents | Disaster victims  (adults) | 1,458 | Longitudinal | Secondary | Regression |
| [63] Han et al. (2021) | Incidents of mass trauma | Infectious disease outbreaks  (COVID-19) | Disaster victims  (adults) | 210 | Cross-sectional | Primary | Structural equation modeling |
| [64] Kang & Kim (2021) | Incidents of mass trauma | Infectious disease outbreaks  (COVID-19) | Community residents  (adults) | 316 | Cross-sectional | Primary | Regression |
| [65] Kang & Moon (2021) | Incidents of mass trauma | Infectious disease outbreaks  (COVID-19) | General population  (20-30’s adults) | 608 | Cross-sectional | Primary | Mediation |
| [66] Kang et al. (2021) | Incidents of mass trauma | Infectious disease outbreaks  (COVID-19) | Disaster victims  (adults) | 107 | Cross-sectional | Primary | Regression |
| [67] Kim & Lee (2021) | Natural | Floods, hurricanes, earthquakes, wildfires | Disaster victims  (adults) | 1,358 | Cross-sectional | Secondary | Regression |
| [68] Kim & Kim (2021) | Natural | Floods, hurricanes, earthquakes, wildfires | Disaster victims  (adults) | 1,122 | Cross-sectional | Secondary | Regression |
| [69] Kim et al. (2021) | Incidents of mass trauma | Infectious disease outbreaks  (COVID-19) | General population  (school parents) | 217 | Cross-sectional | Primary | Correlation |
| [70] Lee D. et al. (2021) | Incidents of mass trauma | Infectious disease outbreaks  (MERS) | Disaster victims  (adults),  Bereaved family | 189 | Cross-sectional | Primary | Regression |
| [71] Lee H. E. et al. (2021) | Man-made | Industrial accidents | Disaster victims  (adults) | 775,537 | Cross-sectional | Secondary | Correlation |
| [72] Lee H. S. et al. (2021) | Incidents of mass trauma | Infectious disease outbreaks  (COVID-19) | Disaster victims  (adults) | 400 | Cross-sectional | Primary | Correlation |
| [73] Lee S. H. et al. (2021) | Incidents of mass trauma | Transport disaster  (sinking of Sewol Ferry) | Disaster victims (survivors),  Bereaved family | 128 | Cross-sectional | Primary | Regression |
| [74] Park S. et al. (2021) | Natural | Floods, hurricanes, earthquakes, wildfires | Disaster victims  (adults),  General population  (adults) | 1,006 | Cross-sectional | Primary | Regression |
| [75] Park J. S. et al. (2021) | Incidents of mass trauma | Infectious disease outbreaks  (COVID-19) | General population  (20’s women) | 122 | Cross-sectional | Primary | Mediation |
| [76] Seong et al. (2021) | Incidents of mass trauma | Infectious disease outbreaks  (COVID-19) | General population  (older adults) | 396 | Cross-sectional | Secondary | Regression |
| [77] Chae & Jung (2022) | Incidents of mass trauma | Infectious disease outbreaks  (COVID-19) | General population  (adults) | 805 | Cross-sectional | Primary | Path |
| [78] Kim & Lee (2022) | Natural | Floods, hurricanes, earthquakes, wildfires | Disaster victims  (adults) | 1,691 | Cross-sectional | Secondary | Regression |
| [79] Kim Y. et al. (2022) | Incidents of mass trauma | Infectious disease outbreaks  (COVID-19) | General population  (adults) | 21,122 | Cross-sectional | Secondary | Difference-in differences |
| [80] Kim E. L. et al. (2022) | Natural | Earthquake  (2017 Pohang earthquake) | Disaster victims  (older adults) | 312 | Cross-sectional | Primary | Structural equation modelling |
| [81] Lee & Byun (2022) | Natural | Floods, hurricanes, earthquakes, wildfires | Disaster victims  (adults) | 3,288 | Cross-sectional | Secondary | Regression |
| [82] Lee & Lee (2022) | Natural | Earthquake  (2016 Gyeongju earthquake) | Disaster victims  (child born after the earthquake) | 4,017 | Cross-sectional | Secondary | Difference-in differences |
| [83] Song & Cho (2022) | Incidents of mass trauma | Infectious disease outbreaks  (COVID-19) | General population  (adults) | 2,001 | Cross-sectional | Primary | Regression |

**REFERENCES**

1.Lee S-H. A study on impact of flood disaster and quality of life among the flood victims. Journal of Korean Academy of Community Health Nursing 2004;15:145-154.

2.Lee I, Ha YS, Kim YA, Kwon YH. PTSD Symptoms in Elementary School Children After Typhoon Rusa. Journal of Korean Academy of Nursing 2004;34:636-645.

3.Chae E-H, Tong Won K, Rhee S-J, Henderson TD. The Impact of Flooding on the Mental Health of Affected People in South Korea. Community Mental Health Journal 2005;41:633-645.

4.Woo J-M, Kang T-Y, Lee J-E. Increasing Risk of Mental Health Problems Among Subway Drivers Experiencing Accidents on the Track. Annals of Occupational and Environmental Medicine 2005;17:36-43.

5.Kim J, Lee D-G, Park I-S, Choi B-C, Kim J-S. Influences of Heat Waves on Daily Mortality in South Korea. Atmosphere 2006;16:269-278.

6.Park SK, Kim DK. A study on factors affecting the mental health among patient with work-related injury. Journal of Rehabilitation Research 2006;10:150-174.

7.Lee D-G, Kim J, Choi B-C. Characteristics of daily mortality due to heat waves in Busan in July 1994. Atmosphere 2007a;17:463-470.

8.Lee EJ, Lim KH, Kim JB, Ryu SY. Posttraumatic Stress Disorder of Survivors in Daegu Subway Fire Accident : A Prospective Study on Predictors. Journal of Korean Neuropsychiatric Association 2007b;46:79-86.

9.Kim K, Kwon S. Psychological Impacts of the Hebei Sprit oil spill event on Taean Residents. ECO 2008;12:83-107.

10.Park S-Y, Kim J-K. Assessment of Quality of Life for Industrial Accident Victims Using SF-36. The Journal of Korean Society of Occupational Therapy 2008;16:73-89.

11.Chang M, Choi K. Mortality for PM10 in Seoul by using Nonlinear Regression Model. Journal of The Korean Data Analysis Society 2009;11:2425-2436.

12.Kim B-M, Park E-K, LeeAn S-Y, Ha M-N, Kim E-J, Kwon H-J, et al. BTEX Exposure and its Health Effects in Pregnant Women Following the Hebei Spirit Oil Spill. Journal of Preventive Medicine and Public Health 2009;42:96-103.

13.Song M-K, Hong Y-C, Cheong H-K, Ha M-N, Kwon H-J, Ha E-H, et al. Psychological Health in Residents Participating in Clean-up Works of Hebei Spirit Oil Spill. Journal of Preventive Medicine and Public Health 2009;42:82-88.

14.Lee C-H, Kang Y-A, Chang K-J, Kim C-H, Hur J-I, Kim J-Y, et al. Acute health effects of the Hebei oil spill on the residents of Taean, Korea. Journal of Preventive Medicine and Public Health 2010;43:166-173.

15.Choi WS, Cho S-A, Kim KY, Cho YS, Koo JW, Kim H-R. The Relationship between the Experience of an Accident and Post Traumatic Stress Disorder in Bus Drivers. Annals of Occupational and Environmental Medicine 2011;23:139-148.

16.Ha KH, Suh M, Kang DR, Kim HC, Shin DC, Kim C. Ambient Particulate Matter and the Risk of Deaths from Cardiovascular and Cerebrovascular Disease. Clinical Hypertension 2011;17:74-83.

17.Lee J-R, Kim D-K. Variation of Vulnerability and Post‐traumatic Stress Disorder of Residents Due to Oil Spill Accident of Hebei Spirit: Analysis of panel data after the accident in Sep, 2008 and Oct, 2010. ECO 2011;15:269-297.

18.Cho Y, Yang S. Peri-Traumatic Dissociation, Post-Traumatic Negative Beliefs, and Poor Social Support As Predictors of Long-Term Psychological Symptoms Following a Natural Disaster. Korean Journal of Clinical Psychology 2013;32:955-979.

19.Kim S, Shin Y, Kim H, Pak H, Ha J. Impacts of typhoon and heavy rain disasters on mortality and infectious diarrhea hospitalization in South Korea. Int J Environ Health Res 2013;23:365-376.

20.Lee KE, Myung HN, Na W, Jang JY. Socio-demographic characteristics and leading causes of death among the casualties of meteorological events compared with all-cause deaths in Korea, 2000-2011. J Prev Med Public Health 2013;46:261-270.

21.Park S-G, Sim M-B. An Empirical Study on Aspect of Regional Conflict in Local Government- With Focus on the 2007 Hebei spirit Oil Spill Incident -. Korean Local Government Review 2013;15:105-133.

22.Bae H-j. Effects of Short-term Exposure to PM10 and PM2.5 on Mortality in Seoul. Journal of Environmental Health Sciences 2014;40:346-354.

23.Lee NY, Cho Y, Lim JY. Effect of Climate Change on Mortality Rate Analysis of Vulnerable Populations. Health and Social Welfare Review 2014;34:456-484.

24.Park J-K, Choi Y-J, Jung W-S. An analysis on the distribution characteristics of PM10 concentration and its relation to the death from Asthma in Seoul, Korea. Journal of Environmental Science International 2015;24:961-968.

25.Shin D-H, Lee N-Y, Cho Y. Analyzing Mortality Rate and Social Costs of Climate Vulnerable Groups caused by Heat Waves in Korea. Journal of Environmental Policy 2015;14:3-32.

26.Yang HJ, Cheong HK, Choi BY, Shin MH, Yim HW, Kim DH, et al. Community mental health status six months after the Sewol ferry disaster in Ansan, Korea. Epidemiol Health 2015;37:e2015046.

27.Choi KH, Lim MH, Ha M, Sohn JN, Kang JW, Choi YH, et al. Psychological Vulnerability of Residents of Communities Affected by the Hebei Spirit Oil Spill. Disaster Med Public Health Prep 2016a;10:51-58.

28.Choi WS, Kim BK, Kim KD, Moon OK, Yeum DM. Impact of the number of painful stimuli on life satisfaction among Korean industrial accident workers completing convalescence: dual mediating effects of self-esteem and sleeping time. Ind Health 2016b;54:460-468.

29.Kim J-W. Exposed Risk of In-house Contracted Workers and Their Absenteeism and Ill-health Symptom Experience Caused by Work-related Accident and Diseases. Korean Journal of Labor Studies 2016;22:1-34.

30.Lee D-H, Kim J-Y, Kang H-S. The Emotional Distress and Fear of Contagion Related to Middle East Respiratory Syndrome(MERS) on General Public in Korea. Korean Journal of Psychology: General 2016;35:355-383.

31.Na W, Lee KE, Myung HN, Jo SN, Jang JY. Incidences of Waterborne and Foodborne Diseases After Meteorologic Disasters in South Korea. Ann Glob Health 2016;82:848-857.

32.Hong KJ, Song KJ, Shin SD, Song SW, Ro YS, Jeong J, et al. Rapid Health Needs Assessment after Typhoons Bolaven and Tembin Using the Public Health Assessment for Emergency Response Toolkit in Paju and Jeju, Korea 2012. J Korean Med Sci 2017;32:1367-1373.

33.Kim JA, Noh SR, Cheong HK, Ha M, Eom SY, Kim H, et al. Urinary oxidative stress biomarkers among local residents measured 6years after the Hebei Spirit oil spill. Sci Total Environ 2017;580:946-952.

34.Kwon H-M, Kim T-H, Choi M-R, Kim B-J, Kim H-W, Song O-S, et al. The Effects of MERS(Middle East Respiratory Syndrome) Event on the Psychosocial Wellbeing of Healthcare Workers and the Public with the Mediating Effect of Resilience. Korean Journal of Psychosomatic Medicine 2017;25:111-119.

35.Lee SH, Kim EJ, Noh JW, Chae JH. Factors Associated with Post-traumatic Stress Symptoms in Students Who Survived 20 Months after the Sewol Ferry Disaster in Korea. J Korean Med Sci 2018a;33:e90.

36.Lee SH, Nam HS, Kim HB, Kim EJ, Noh JW, Chae JH. Factors Associated with Complicated Grief in Students Who Survived the Sewol Ferry Disaster in South Korea. Psychiatry Investig 2018b;15:254-260.

37.Lim HS, Sim K. The Effects of Life Changes on Post-Traumatic Stress Disorder after Disasters. STRESS 2018;26:319-326.

38.Min M-K, Joo H-S, Ahn H-N. Psychosocial Factors Influential to the Mental Health of the Public Indirectly Affected by the 9/12 Gyeong-ju Earthquake: Focusing on Individual Resilience, Social Support, Social Capital, and Public Trust. Korean Journal of Counseling 2018;19:93-116.

39.Yang HJ, Kim G, Lee K, Lee J, Cheong HK, Choi BY, et al. Changes in the levels of depressive symptoms and anxiety in Ansan city after the 2014 Sewol ferry disaster. J Affect Disord 2018;241:110-116.

40.Cho MS. Factors associated with Quality of Life among Disaster Victims: An Analysis of the 3rd Nationwide Panel Survey of Disaster Victims. Journal of Korean Academy of Community Health Nursing 2019;30:217-225.

41.Han KM, Park JY, Park HE, An SR, Lee EH, Yoon HK, et al. Social support moderates association between posttraumatic growth and trauma-related psychopathologies among victims of the Sewol Ferry Disaster. Psychiatry Res 2019;272:507-514.

42.Jeon SY, Seo HY, Kim YH. A Retrospective Analysis on the Psychopathology of Victims in Jecheon Fire Accident. Crisisonomy 2019;15:97-111.

43.Kim H, Kim M. The Impact of Disaster Victim’s Social Support on Quality of Life: Mediating Effect of Resilience. The Journal of Humanities and Social Science (HSS21) 2019;10:841-854.

44.Kim JS, Lee SY. The Effect of Psychosocial Factors on Post Traumatic Stress Disorder (PTSD) Groups of Natural Disaster Victims. The Korean Journal of Health Psychology 2019;24:669-693.

45.Kim I, Oh WO. Predictors of Posttraumatic Growth Among University Students Who Experienced a Recent Earthquake. Issues Ment Health Nurs 2019;40:176-184.

46.Kim J. Post-Disaster Inequality Factors on Depression and Anxiety among Elderly Victims in South Korea. Crisisonomy 2019;15:27-41.

47.Kim S, Cho S, Song Y. Moderating Effects of Social Support and Social Participation of the Relationship between Depression and Quality of Life of Elderly Victims of Disaster. Korean Journal of Gerontological Social Welfare 2019;74:9-34.

48.Noh JW, Kim KB, Lee Y, Lee JH, Kim EJ, Lee SH. Factors associated with health-related quality of life (HRQOL) in students who survived the Sewol ferry disaster in South Korea. J Affect Disord 2019;249:223-225.

49.Sim K. The Psychiatric Comorbidity in Disaster Victims with PTSD: Comorbidity Rates, Severity of Psychological Disorders and Factors Predicting Comorbidities. STRESS 2019;27:259-267.

50.Choi S-S. The Effects of Conflict Experience and Social Participation on Depression after Disaster in Elderly. Social Welfare Policy and Practice 2020;6:45-77.

51.Kim DH, Kim HJ. The Relationship Between Posttraumatic Stress Response and The Internalization of Problems in Disaster Victims: A Yearlong Follow-up Based on The Diathesis-stress Model and Resilience Theory. Journal of Rehabilitation Psychology 2020;27:21-42.

52.Kim YR, Lee W. The Effects of Social Support on the Quality of Life among Disaster Victims in One-person Households. Crisisonomy 2020;16:1-15.

53.Kim YR, Oh SM. The Effect of Community Resilience on Post-Traumatic Stress Disorders (PTSD) in Disaster Victims. Crisisonomy 2020;16:73-90.

54.Kim SY. An Analysis of Factors Influencing Post-traumatic Stress Disorder (PTSD) - Difference by Disaster Information -. Crisisonomy 2020a;16:65-78.

55.Kim S. A Study on Factors Affecting the Resilience of Disaster Victims -Focusing on Comparison Between Single and Multi-person Households. The Journal of Humanities and Social Science (HSS21) 2020b;11:95-108.

56.Lee N, Lee JH, Yoo S, Sim M. The secondary stress factors influencing the onset of mental disorders following a disaster. Korean Journal of Social and Personality Psychology 2020a;34:19-36.

57.Lee D-H, Kim Y-J, Lee D-H, Hwang H-H, Nam S-K, Kim J-Y. The Influence of Public Fear, and Psycho-social Experiences during the Coronavirus Disease 2019(COVID-19) Pandemic on Depression and Anxiety in South Korea. The Korean Journal of Counseling and Psychotherapy 2020b;32:2119-2156.

58.Lim J, Chang M-S, Kim K. A Study on the Psychological Trauma, Safety Climate, Safety Thought, and Safety Behavior of Workers who Experienced Fatal Workplace Accidents. The Korean Journal of Health Psychology 2020;25:1077-1095.

59.Park J, Chae Y. Analysis of heat-related illness and excess mortality by heat waves in South Korea in 2018. Journal of the Korean Geographical Society 2020;55:391-408.

60.Song Y-J, Kwon H-M, Ryu J-S. Quality of Life and Influencing Factors of Elderly Victims of Disaster. Journal of Wellness (KSW) 2020;15:61-71.

61.Song Y, Choi S. The Factors Influencing Post Disaster Social Adjustment of the Elderly in Disaster Vulnerable Group. The Journal of Humanities and Social Science (HSS21) 2020;11:1769-1780.

62.Bae SW, Jeong I, Yoon JH, Lee SW, Kim TH, Won JU. Relationship between workers' return to work, job retention and income in industrial accidents in Korea: a longitudinal study. BMJ Open 2021;11:e039948.

63.Han J-S, Choi J-H, Lee S-O, Kim Y-R, Kim S-S. The Effect of Empathy on Anxiety and Depression in COVID-19 Disaster : through Risk Perception and Indirect Trauma. The Journal of the Korea Contents Association 2021;21:609-625.

64.Kang HS, Kim BN. The Role of Event-Related Rumination and Perceived Social Support on Psychological Distress during the COVID-19 Pandemic: Results from Greater Daegu Region in South Korea. Psychiatry Investig 2021;18:392-399.

65.Kang S-J, Moon J-Y. Does Drinking in Social Disasters Affect Community Conflicts?- For young people in their 20s and 30s. -. Studies on Life and Culture 2021;60:21-49.

66.Kang E, Lee SY, Kim MS, Jung H, Kim KH, Kim KN, et al. The Psychological Burden of COVID-19 Stigma: Evaluation of the Mental Health of Isolated Mild Condition COVID-19 Patients. J Korean Med Sci 2021;36:e33.

67.Kim Y, Lee H. Sleep Problems among Disaster Victims: A Long-Term Survey on the Life Changes of Disaster Victims in Korea. Int J Environ Res Public Health 2021;18

68.Kim M-S, Kim H-S. The Effect of Disaster Victim’s Community Resillience on Social Adaptation. Social Welfare Policy and Practice 2021;7:149-181.

69.Kim SJ, Lee S, Han H, Jung J, Yang SJ, Shin Y. Parental Mental Health and Children's Behaviors and Media Usage during COVID-19-Related School Closures. J Korean Med Sci 2021;36:e184.

70.Lee D, Lee JH, Jeon K, Lee N, Sim M. Psychological Responses Among Korean Middle East Respiratory Syndrome Survivors and Bereaved Families. Disaster Med Public Health Prep 2021a;16:1077-1082.

71.Lee HE, Kim I, Kim MH, Kawachi I. Increased risk of suicide after occupational injury in Korea. Occup Environ Med 2021b;78:43-45.

72.Lee HS, Dean D, Baxter T, Griffith T, Park S. Deterioration of mental health despite successful control of the COVID-19 pandemic in South Korea. Psychiatry Res 2021c;295:113570.

73.Lee SH, Noh JW, Kim KB, Kim EJ, Oh J, Chae JH. Factors associated with post-traumatic stress disorder among bereaved family members and surviving students two and half years after the Sewol ferry accident in South Korea. Psychiatry Res 2021d;296:113666.

74.Park S, Kim S, Kim GU, Noh D. Effects of social support on mental health outcomes in disasters: A cross-sectional study. Nurs Health Sci 2021a;23:456-465.

75.Park JS, Kim HY, Lee SJ. COVID-19 related anxiety among young women and its influence on government and community trust: the mediating effect between Corona Blue and Corona Red. Korean Society and Public Administration 2021b;32:95-117.

76.Seong K, Kim S, Moon J. Effects of Life Changes Due to COVID-19 on Depression in the Elderly. Social Work Practice & Research 2021;18:99-129.

77.Chae J, Jung EH. Thinking about Social Consequences of COVID-19 Influenced Preventive Intention: The Case of South Korea. Health Commun 2023;38:1563-1571.

78.Kim JS, Lee SY. Protective and Risk Factors That Influence PTSS and Comorbidity of Natural Disaster Victims. The Korean Journal of Health Psychology 2022;27:1-26.

79.Kim Y, Kwon JA, Kim KY. The Effect of Covid-19 Pandemic on Individual Mental Health: Focusing on Comparison of Different Life Cycles in Adulthood. Journal of Critical Social Policy 2022a:7-37.

80.Kim EM, Kim GS, Kim H, Park CG, Lee O, Pfefferbaum B. Health-related quality of life among older adults who experienced the Pohang earthquake in South Korea: A cross-sectional survey. Health Qual Life Outcomes 2022b;20:37.

81.Lee G, Byun B. The Effect of Quality of Life of Disaster Victims on Resilience: Focusing on the Moderating Effect of Social Support. Journal of Environmental Policy and Administration 2022;30:255-280.

82.Lee Y, Lee T-J. Consequences of In Utero Exposure to the Gyeongju Earthquake. Health and Social Welfare Review 2022;42:146-165.

83.Song HJ, Cho YJ. Determinants of Subjective Well-being Under the COVID-19 Experience: A Comparison Between Generations. Journal of Korean Social Welfare Administration 2022;24:119-149.
